# Loss of *Nlrp3* Does Not Protect Mice from Western Diet-Induced Adipose Tissue Inflammation and Glucose Intolerance

**DOI:** 10.1371/journal.pone.0161939

**Published:** 2016-09-01

**Authors:** Rebecca E. Ringling, Michelle L. Gastecki, Makenzie L. Woodford, Kelly J. Lum-Naihe, Ryan W. Grant, Lakshmi Pulakat, Victoria J. Vieira-Potter, Jaume Padilla

**Affiliations:** 1 Department of Nutrition and Exercise Physiology, University of Missouri, Columbia, Missouri, United States of America; 2 Department of Medical Pharmacology and Physiology, University of Missouri, Columbia, Missouri, United States of America; 3 Department of Nutrition Science, Purdue University, West Lafayette, Indiana, United States of America; 4 Department of Medicine, University of Missouri, Columbia, Missouri, United States of America; 5 Research Service, Harry S Truman Memorial Veterans Affairs Hospital, Columbia, Missouri, United States of America; 6 Dalton Cardiovascular Research Center, University of Missouri, Columbia, Missouri, United States of America; 7 Department of Child Health, University of Missouri, Columbia, Missouri, United States of America; Monash University, AUSTRALIA

## Abstract

We tested the hypothesis that loss of *Nlrp3* would protect mice from Western diet-induced adipose tissue (AT) inflammation and associated glucose intolerance and cardiovascular complications. Five-week old C57BL6J wild-type (WT) and *Nlrp3* knockout (*Nlrp3*^-/-^) mice were randomized to either a control diet (10% kcal from fat) or Western diet (45% kcal from fat and 1% cholesterol) for 24 weeks (n = 8/group). Contrary to our hypothesis that obesity-mediated white AT inflammation is *Nlrp3*-dependent, we found that Western diet-induced expression of AT inflammatory markers (i.e., *Cd68*, *Cd11c*, *Emr1*, *Itgam*, *Lgals*, *Il18*, *Mcp1*, *Tnf*, *Ccr2*, *Ccl5* mRNAs, and Mac-2 protein) were not accompanied by increased caspase-1 cleavage, a hallmark feature of NLRP3 inflammasome activation. Furthermore, *Nlrp3* null mice were not protected from Western diet-induced white or brown AT inflammation. Although Western diet promoted glucose intolerance in both WT and *Nlrp3*^-/-^ mice, *Nlrp3*^-/-^ mice were protected from Western diet-induced aortic stiffening. Additionally, *Nlrp3*^-/-^ mice exhibited smaller cardiomyocytes and reduced cardiac fibrosis, independent of diet. Collectively, these findings suggest that presence of the *Nlrp3* gene is not required for Western diet-induced AT inflammation and/or glucose intolerance; yet *Nlrp3* appears to play a role in potentiating arterial stiffening, cardiac hypertrophy and fibrosis.

## Introduction

Current evidence implicates visceral white adipose tissue (AT) inflammation, or visceral adiposopathy [1], as a causal link between obesity and cardiometabolic complications [2–5]. However, the molecular mechanisms by which obesity promotes AT inflammation remain poorly understood. Adipocyte hypertrophy in response to caloric excess leads to infiltration of immune cells such as macrophages and T lymphocytes into AT, which can cross-activate one another, hence perpetuating the secretion of inflammatory cytokines from AT [4, 6–8]. AT is viewed as an active immunological organ that controls whole-body metabolism and cardiovascular function through endocrine mechanisms [2–4].

Immune cells are equipped with the NOD-like receptor family, pyrin domain containing 3 (NLRP3) inflammasome, which is an intracellular multi-protein complex vital for the protection against numerous pathogen-derived factors [9–13]. It consists of a NOD-like receptor, the apoptosis-associated speck-like protein containing a Card (Pycard), and pro-caspase-1. The NLRP3 inflammasome can also sense metabolic “danger signals” originating from Western diet and obesity such as glucose, cholesterol, free fatty acids, uric acid, and reactive oxygen species. Activation of the NLRP3 inflammasome causes caspase-1 cleavage and subsequent secretion of pro-inflammatory cytokines, IL-1β and IL-18, and it is thought to stimulate classic pro-inflammatory (M1) polarization of macrophages [9–13]. As such, it is conceivable that obesity-induced activation of NLRP3 promotes activation and infiltration of macrophages into AT leading to increased expression of inflammatory genes. However, whether loss of *Nlrp3* would protect mice from Western diet-induced AT inflammation remains largely unknown. Furthermore, given the known role of AT inflammation in instigating metabolic and cardiovascular complications [2, 3], we also hypothesized that deletion of *Nlrp3* would alleviate Western diet-induced glucose intolerance, arterial stiffening, and cardiac hypertrophy and fibrosis.

## Materials and Methods

### Experimental Design

Male C57BL6J wild-type (WT, n = 16; Stock 000664) and *Nlrp3* knockout (*Nlrp3*^-/-^, n = 16; B6.129S6-Nlrp3<tm1Bhk>/J; Stock 021302) mice on a C57BL6J background were purchased from Jackson Laboratory (Bar Harbor, ME). WT and *Nlrp3*^-/-^ mice were not littermates. At 5 weeks of age, mice were randomized to either a control diet or Western diet (n = 8/group) *ad libitum* for 24 weeks. Control diet (3.85 kcal/g of food) contained 10% kcal fat, 70% kcal carbohydrate, and 20% kcal protein, with 3.5% kcal sucrose (D12110704; Research Diets Inc.). Western diet (4.68 kcal/g of food) contained 44.9% kcal fat, 35.1% kcal carbohydrate, and 20% kcal protein, with 1% cholesterol and 17% kcal sucrose (D09071604; Research Diets Inc.). All mice were pair-housed and kept at 25°C with a light cycle from 0700 to 1900 and a dark cycle from 1900 to 0700 under conventional (i.e., non-specific pathogen free) animal housing facilities. At 29 weeks of age, mice were euthanized via CO_2_ inhalation following a 5-hour fast. Samples were harvested and stored at -80°C until analysis. Male mice were used in this study because they are more susceptible to Western diet-induced AT inflammation [14]. All procedures were approved in advance by the University of Missouri Institutional Animal Care and Use Committee.

### Total Energy Expenditure

Using a metabolic monitoring system (Promethion, Sable Systems Int., Las Vegas, NV), total energy expenditure during the 12-hour light and 12-hour dark cycles were determined by monitoring oxygen consumption and carbon dioxide production over a 3-day period, as previously described [15]. Total energy expenditure was calculated using body weight as covariate according to current recommendations [16]. These measurements were performed at 15 weeks of age.

### Glucose Tolerance Testing

Glucose tolerance tests were performed at 15 and 25 weeks of age. Briefly, after a 5-hour fast, blood glucose was measured from the tail vein. The tail was nicked and blood was sampled by a hand-held glucometer (Alpha Trak, Abbott Labs). A baseline measure of blood glucose was taken prior to giving a sterile solution of 50% dextrose (2g/kg body weight (BW)) via intraperitoneal injection, as previously performed. Glucose measures were taken 15, 30, 45, 60 and 120 minutes after the glucose injection. Glucose area under curve (AUC) from baseline was calculated.

### Aortic Stiffness by In Vivo Pulse Wave Velocity

Doppler ultrasound (Indus Mouse Doppler System, Webster, TX) was used as previously described [17] to evaluate pulse wave velocity (PWV), the gold standard technique for *in vivo* determination of arterial stiffness. Prior to sacrifice, isoflurane-anesthetized mice (1.75% in 100% oxygen stream) were placed supine on a heating board and legs secured to ECG electrodes. Determination of PWV is based on the transit time method calculated as the difference in arrival times of a Doppler pulse wave at two locations along the aorta a known distance apart. Each of the pulse wave arrival times is measured as the time from the peak of the ECG R-wave to the leading foot of the pulse wave at which time velocity begins to rise at the start of systole. The distance between the two locations along the aorta is measured with a ruler and divided by transit time. Data are expressed in cm/s. Velocity waveforms were acquired at the aortic arch followed immediately by measurement at the descending aorta proximal to the iliac bifurcation.

### Fasting Blood Parameters

Blood was obtained at time of sacrifice following a 5-hour fast. Plasma glucose, cholesterol, triglycerides, alanine aminotransferase (ALT), aspartate aminotransferase (AST), and non-esterified fatty acids (NEFA) assays were performed by a commercial laboratory (Comparative Clinical Pathology Services, Columbia, MO) on an Olympus AU680 automated chemistry analyzer (Beckman-Coulter, Brea, CA) using assays according to manufacturer’s guidelines. Plasma insulin concentrations were determined using a commercially available, mouse-specific ELISA (Alpco Diagnostics, Salem, NH). Plasma concentration of IL-18 was assessed using a mouse specific ELISA (Medical and Biological Laboratories, product #7625). Plasma concentrations of IL1β, MCP-1, TNF-α, and IL-6 were assessed using a mouse-specific multiplex cytokine assay (Millipore Milliplex; Billerica, MA, USA) on a MAGPIX instrument (Luminex Technologies; Luminex Corp., Austin, TX, USA) according to the manufacturer’s instructions. Unfortunately, plasma levels of IL1β, MCP-1, and TNF-α were below the detection limit of this assay, thus only IL-6 data are presented. The whole blood samples were analyzed for HbA1c using a boronate affinity HPLC method, Trinity ultra2 (Kansas City, MO), as previously described [18].

### Histological Assessments

Formalin-fixed retroperitoneal AT and liver samples were processed through paraffin embedment, sectioned at 5 μm, stained with hematoxylin and eosin (H&E) for morphometric determinations. AT samples were also stained for Mac-2 (CL8942AP, Cedarlane), a macrophage marker. Sections were evaluated via an Olympus BX34 photomicroscope (Olympus, Melville, NY) and images were taken via an Olympus SC30 Optical Microscope Accessory CMOS color camera. Adipocyte size was calculated based on 100 adipocytes/animal from three 10x fields of view, as performed previously [18]. Briefly, cross-sectional areas of the adipocytes were obtained from perimeter tracings using Image J software (NIH public domain; National Institutes of Health, Bethesda, MD). Objective quantification of macrophage infiltration was done by determining the positive Mac-2 stained area per 10x field of view using Image J software. The average of three 10x fields of view was used per animal. Cardiomyocyte hypertrophy was measured as previously described [19, 20]. Briefly, 5μm formalin-fixed, paraffin-embedded cardiac sections were stained with WGA conjugated to Alexa Fluor 647 (Life Technologies). Two to three images per section were captured using a Leica DMI4000B inverted confocal microscope at 40x. The smallest transverse widths (cardiomyocyte diameter) were measured of ~45–50 cross-sections of cardiomyocytes from each animal and averaged, using Image J Software. Cardiac fibrosis was determined from trichrome blue-stained slides. The positive trichrome blue-stained area per 10x field of view was manually traced using Image J software. The average of three 10x fields of view was used per animal. All histological procedures and analysis were performed by an investigator who was blinded to the groups.

### Liver Triglycerides

Hepatic triacylglycerol (TG) concentration was determined using a commercially available kit (Wako L-Type TG M; Wako Pure Chemical Industries, Osaka, Japan). A BioTek uQUANT microplate spectrophotometer (Biotek Instruments, Winooski, VT) was used to analyze the absorbance set at a wavelength of 582 nm. Data are expressed as milligrams TG/gram of liver (wet weight), as described previously [18].

### Gene Expression in Adipose Tissue and Stromal Vascular Cells Isolated from Adipose Tissue

Assessment of gene expression via real-time RT-PCR was performed in retroperitoneal white AT, interscapular brown AT, and the stromal vascular cell fraction freshly isolated from epididymal white AT. Stromal vascular cells were isolated via collagenase digestion as previously described [21]. All samples were homogenized and lysed in TRIzol solution using a tissue homogenizer (TissueLyser LT, Qiagen, Valencia, CA). Total RNA was isolated according to the Qiagen’s RNeasy lipid tissue protocol and assayed using a Nanodrop spectrophotometer (Thermo Scientific, Wilmington, DE) to assess purity and concentration. The MU DNA Core confirmed our RNA extraction protocol produces optimal RNA integrity (all RQN’s>8.0). First-strand cDNA was synthesized from total RNA using the High Capacity cDNA Reverse Transcription kit (Applied Biosystems, Carlsbad, CA). Quantitative real-time PCR was performed as previously described using the ABI StepOne Plus sequence detection system (Applied Biosystems). Primer sequences were designed using the NCBI Primer Design tool and have been previously published [18]. All primers were purchased from IDT (Coralville, IA). A 20 μL reaction mixture containing 10 μL iTaq UniverSYBR Green SMX (BioRad, Hercules, CA) and the appropriate concentrations of gene-specific primers plus 4 μL of cDNA template were loaded in a single well of a 96-well plate. All PCR reactions were performed in duplicate under thermal conditions as follows: 95°C for 10 min, followed by 40 cycles of 95°C for 15 s and 60°C for 45 s. A dissociation melt curve analysis was performed to verify the specificity of the PCR products. *Gapdh* was used as house-keeping control gene. *Gapdh* cycle threshold (CT) was not different among the groups of animals. mRNA expression values are presented as 2^ΔCT^ whereby ΔCT = *Gapdh* CT—gene of interest CT. mRNA levels were normalized to the WT control diet group.

### Western Blotting

Triton X-100 tissue lysates were used to produce Western blot-ready Laemmli samples. Protein samples (10 μg/lane) were separated by SDS-PAGE, transferred to polyvinylidene difluoride membranes, and probed with Mac-2 (#12733, 1:1000, Cell Signaling) and pro-caspase-1 and p10 caspase-1 (SC-514, 1:500, Santa Cruz). Intensity of individual protein bands were quantified using FluoroChem HD2 (AlphaView, version 3.4.0.0), and expressed as ratio to control band GAPDH (#5174, 1:1000, Cell Signaling) or β-tubulin (#2146, 1:1000, Cell Signaling).

### Statistical Analysis

A 2x2 (diet x genotype) analysis of variance (ANOVA) was used to evaluate the effects of Western diet and *Nlrp3* deficiency on all dependent variables. Main effects and diet by genotype interaction were examined, and Fisher’s LSD post-hoc test was used for pair-wise comparisons. All data are presented as mean ± standard error (SE). For all statistical tests, the alpha level was set at 0.05. All statistical analyses were performed with SPSS V23.0.

## Results

**Fig 1:**
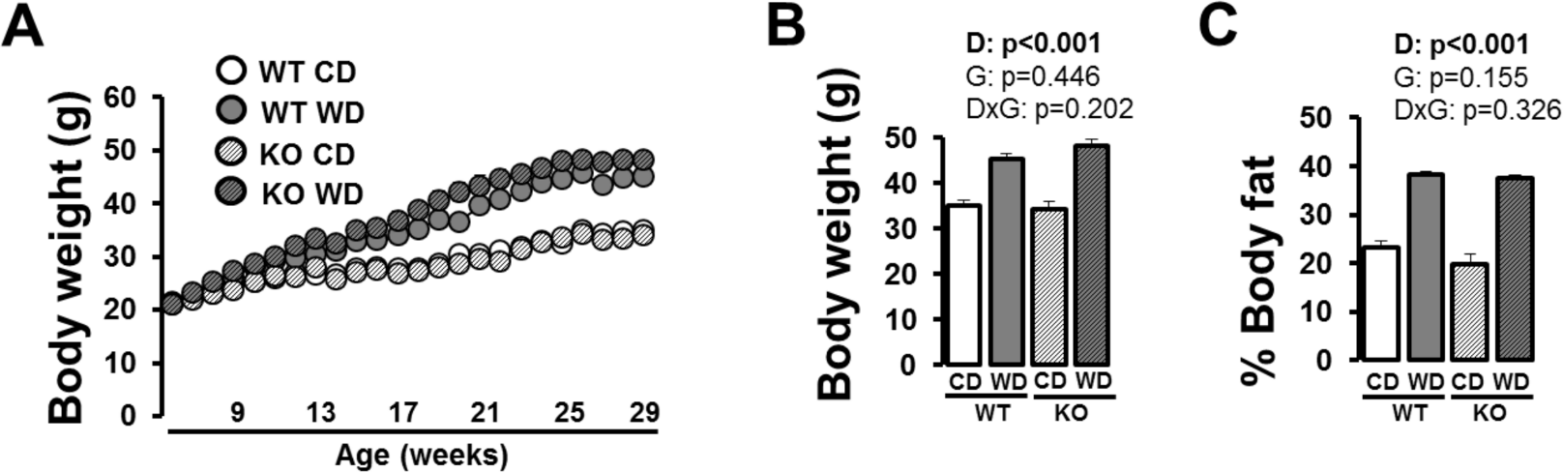
Body weight and composition in WT and *Nlrp3*^-/-^ mice fed a control diet versus Western diet. (A) Weekly body weights; (B) final body weight; (C) final % body fat. Data are expressed as means ± SE. WT, wild-type; KO, *Nlrp3* knockout, CD, control diet; WD, Western diet; D, main effect of diet; G, main effect of genotype; DxG, diet by genotype interaction. Significant p values (<0.05) are highlighted in bold.

As shown in Fig 1, independent of genotype, mice fed a Western diet were heavier and had greater adiposity compared to control diet-fed mice (p<0.05). A trend was observed for Western diet-fed *Nlrp3*^-/-^ mice to have greater body weights than WT counterparts, however, this difference did not reach statistical significance (p = 0.144). Western diet-fed *Nlrp3*^-/-^ mice consumed more kilocalories per week than WT over the 2-week period of food intake assessment (Table 1, p<0.05). No significant effects of genotype were observed in total energy expenditure (using body weight as covariate [16]) during the light cycle (WT control diet = 3.5±0.23 vs. *Nlrp3*^-/-^ control diet = 3.74±0.23 kcal/h, p = 0.475; WT Western diet = 4.23±0.07 vs. *Nlrp3*^-/-^ Western diet = 4.19±0.08 kcal/h, p = 0.756) or dark cycle (WT control diet = 5.24±0.19 vs. *Nlrp3*^-/-^ control diet = 4.78±0.17 kcal/h, p = 0.109; WT Western diet = 5.20±0.12 vs. *Nlrp3*^-/-^ Western diet = 5.13±0.13 kcal/h, p = 0.681). Independent of genotype, compared to control-fed mice, Western diet-fed mice had greater plasma total cholesterol, low density lipoprotein, high density lipoprotein, alanine aminotransferase, aspartate aminotransferase, insulin, and HOMA-IR (Table 1, p<0.05). HbA1c was increased with Western diet in both WT and knockout mice (p<0.05); however, knockout mice exhibited slightly but significantly lower levels of HbA1c compared to WT mice, independent of diet (Table 1, p<0.05). Notably, plasma IL-18, a hallmark of NLRP3 inflammasome activation, was reduced in *Nlrp3*^-/-^ mice fed control and Western diets (Table 1, p<0.05).

**Table 1:** Animal characteristics.

Variable	Wild-type		*Nlrp3*^-/-^		Two-Way ANOVA		
	Control diet	Western diet	Control diet	Western diet	Diet effect	Genotype effect	Interaction
**Kcal consumed/week**	85.5±3.5	90.6±2.8	80.5±3.6	99.7±3.1	**P = 0.001**	P = 0.544	P = 0.041
**Retroperitoneal AT weight (g)**	0.21±0.03	0.46±0.03	0.20±0.04	0.43±0.03	**P<0.001**	P = 0.503	P = 0.768
**Interscapular brown AT weight (g)**	0.18±0.03	0.24±0.02	0.17±0.02	0.27±0.03	**P = 0.001**	P = 0.479	P = 0.419
**Heart weight/body weight (%)**	0.44±0.01	0.34±0.01	0.44±0.02	0.34±0.01	**P<0.001**	P = 0.962	P = 0.782
**Total cholesterol, mg/dL**	114.3±7.7	239.5±22.1	108.5±8.4	249.9±29.8	**p<0.001**	p = 0.910	p = 0.687
**LDL cholesterol, mg/dL**	4.7±0.4	21.5±3.3	4.3±0.3	27.0±5.7	**p<0.001**	p = 0.454	p = 0.384
**HDL cholesterol, mg/dL**	58.2±3.6	98.5±6.1	58.4±3.6	94.4±6.1	**p<0.001**	p = 0.712	p = 0.682
**Triglycerides, mg/dL**	106.7±9.2	112.6±38.1	100.5±15.8	89.7±13.1	p = 0.921	p = 0.554	p = 0.732
**NEFA, mmol/L**	0.75±0.06	0.39±0.03	0.83±0.06	0.40±0.02	**p<0.001**	p = 0.343	p = 0.527
**ALT, U/L**	165.2±81.0	504.9±133.9	78.3±16.4	748.3±149.1	**p<0.001**	p = 0.497	p = 0.158
**AST, U/L**	249.4±92.6	319.1±61.0	175.5±18.0	397.6±71.6	**p = 0.026**	p = 0.971	p = 0.228
**Insulin, ng/mL**	1.2±0.3	1.8±0.3	1.1±0.2	2.7±0.6	**p = 0.004**	p = 0.269	p = 0.224
**Glucose, mg/dL**	269.8±31.7	320.3±28.3	265.5±23.2	320.7±27.8	p = 0.069	p = 0.945	p = 0.932
**HOMA-IR**	24.0±7.2	42.4±7.0	23.3±4.9	64.9±15.2	**p = 0.003**	p = 0.251	p = 0.222
**Hba1c, %**	4.42±0.06	4.68±0.04	4.19±0.05	4.59±0.05	**p<0.001**	**p = 0.004**	p = 0.181
**IL-18, pg/mL**	296.9±48.1	302.5±31.6	249.6±27.8	203.0±19.1	p = 0.530	**P = 0.031**	p = 0.424
**IL-6, pg/mL**	148.5±87.1	161.5±91.7	51.1±24.2	41.1±12.3	p = 0.982	p = 0.103	p = 0.860

Abbreviations: LDL, low density lipoprotein; HDL, high density lipoprotein; NEFA; non-esterified fatty acids; ALT, alanine aminotransferase; AST, aspartate aminotransferase; HOMA-IR, homeostatic model assessment insulin resistance index. Data are expressed as means ± SE.D. Significant p values (<0.05) are highlighted in bold.

**Fig 2:**
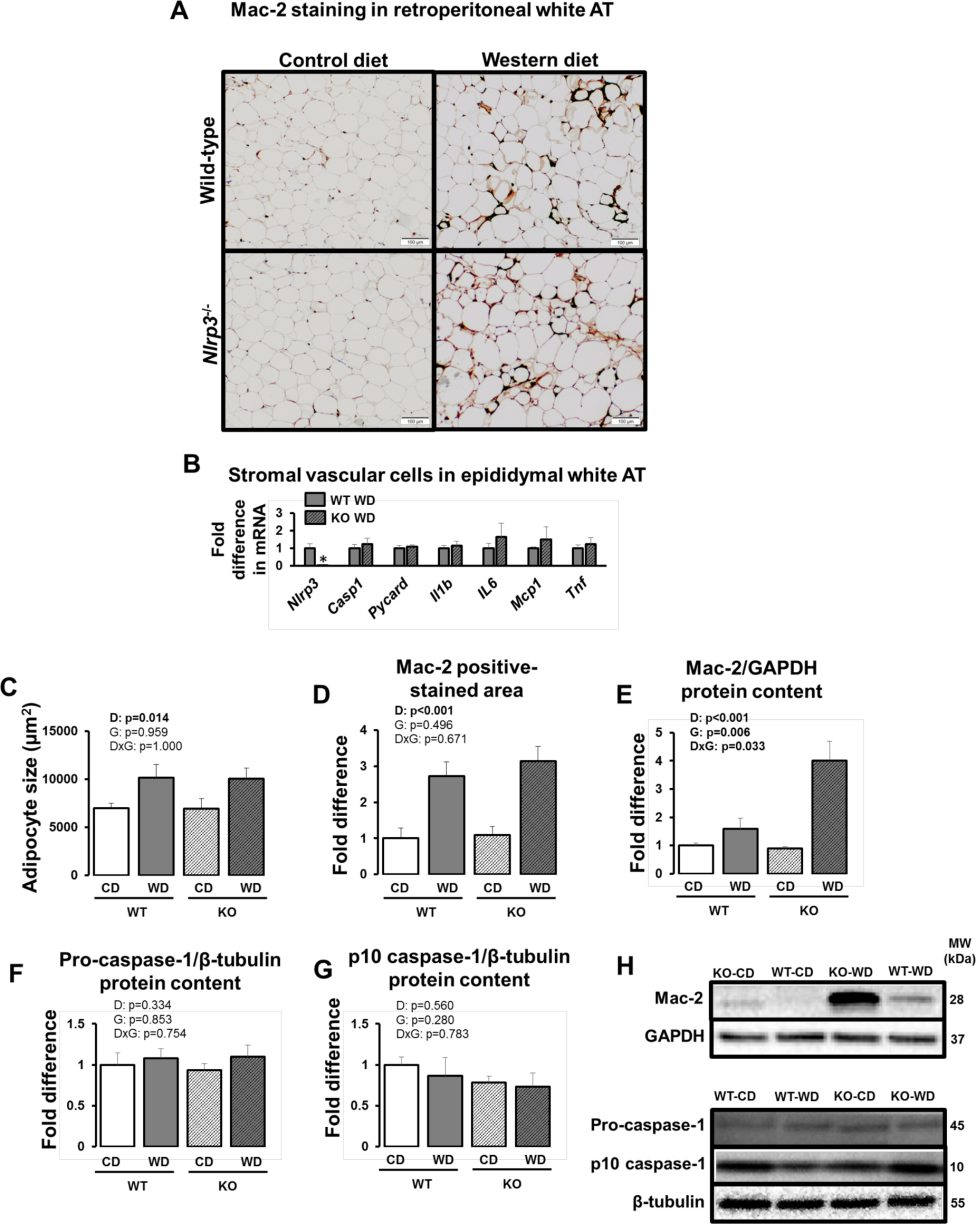
Visceral white AT characterization in WT and *Nlrp3*^-/-^ mice fed a control diet versus Western diet. (A) Representative immunohistochemical 10X images of retroperitoneal white AT stained for Mac-2; (B) gene expression in stromal vascular cells isolated from epididymal white AT; (C) average adipocyte size in retroperitoneal white AT; (D) Mac-2 positive immunostained area in retroperitoneal white AT; (E) protein content of Mac-2 via Western blotting in retroperitoneal white AT; (F) protein content of pro-caspase-1 via Western blotting in retroperitoneal white AT; (G) protein content of p10 caspase-1 (cleavage) via Western blotting in retroperitoneal white AT; (H) representative Western blot bans. Data are expressed as means ± SE. WT, wild-type; KO, *Nlrp3* knockout, CD, control diet; WD, Western diet; D, main effect of diet; G, main effect of genotype; DxG, diet by genotype interaction. Significant p values (<0.05) are highlighted in bold. *denotes p<0.05 in panel B.

**Fig 3:**
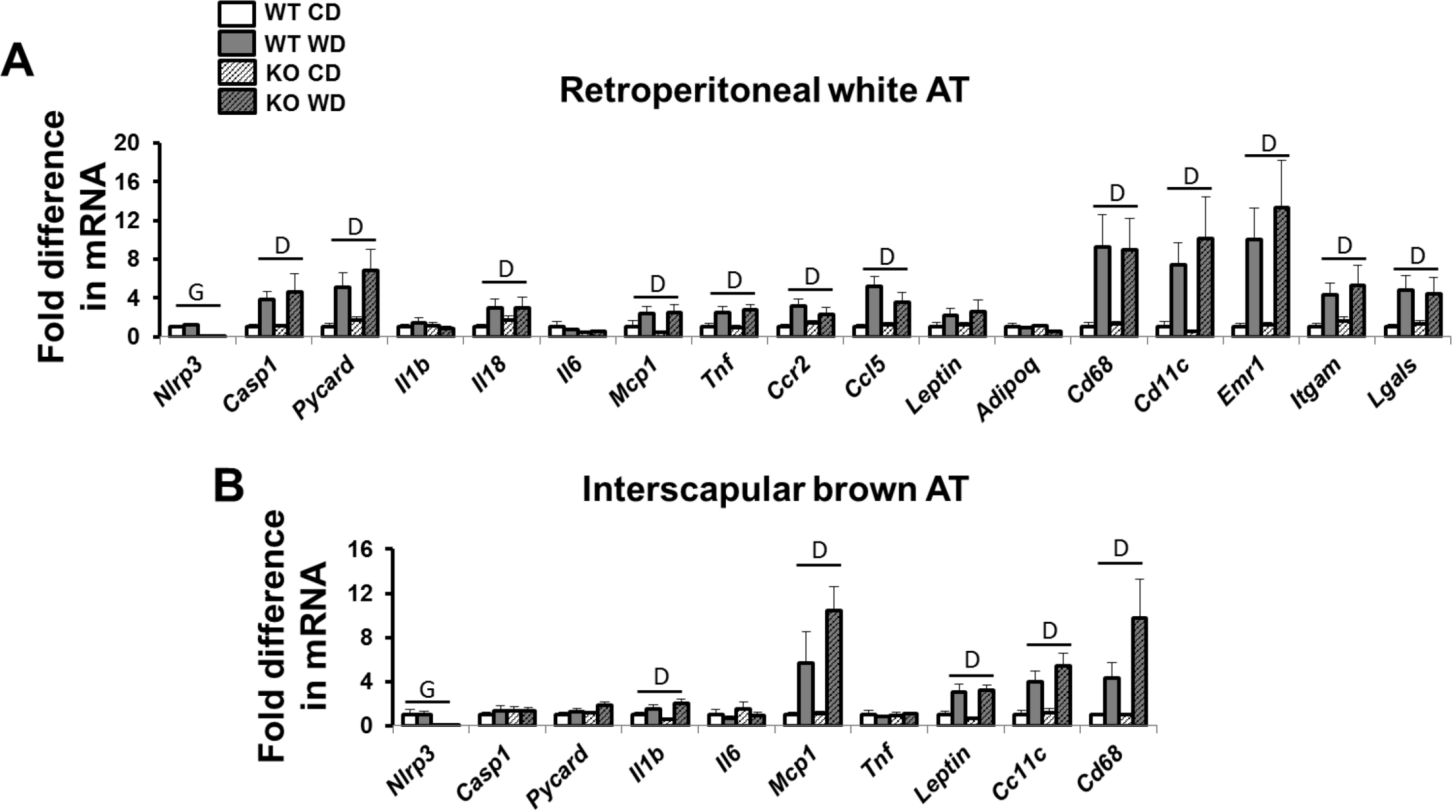
AT gene expression in WT and *Nlrp3*^-/-^ mice fed a control diet versus Western diet. (A) Visceral white (i.e., retroperitoneal) AT; (B) brown (i.e., interscapular) AT. Data are expressed as means ± SE. WT, wild-type; KO, *Nlrp3* knockout, CD, control diet; WD, Western diet; D, main effect of diet (p<0.05); G, main effect of genotype (p<0.05). No significant interactions were found.

As illustrated in Fig 2A and 2C, adipocyte size in retroperitoneal white AT was increased with Western diet, independent of genotype (p<0.05). Similarly, levels of Mac-2 expression, examined by immunohistochemistry (Fig 2A and 2D) and Western blotting (Fig 2E), were increased with Western diet in both WT and knockout mice (p<0.05). In addition, ablation of *Nlrp3* did not alter expression of other components of NLRP3 inflammasome and inflammatory cytokines in stromal vascular cells isolated from white AT of Western diet-fed mice (Fig 2B). As confirmation of our model, *Nlrp3* mRNA levels were undetectable in *Nlrp3* null mice. Fig 3 summarizes gene expression data in retroperitoneal white AT (panel A) and interscapular brown AT (panel B). Western diet significantly increased white AT expression of the components of the NLRP3 inflammasome (*Nlrp3*, *Pycard*, *Caspase-1* mRNAs), markers of macrophage infiltration (i.e., *Cd68*, *Cd11c*, *Emr1*, *Itgm*, *and Lgals* mRNAs) and inflammation (i.e., *Il18*, *Mcp1*, *Tnfα*, *Ccr2*, *Ccl5* mRNAs). However, contrary to our hypothesis, these inflammatory effects were not accompanied by increased caspase-1 cleavage (Fig 2G; p>0.05). Furthermore, markers of white AT inflammation were not reduced in *Nlrp3*^-/-^ relative to WT mice (Fig 3A; p>0.05). Also in both genotypes, Western diet led to increased expression of *Il1β*, *Mcp1*, *Leptin*, *Cc11c*, and *Cd68* mRNAs (p<0.05) in brown AT.

**Fig 4:**
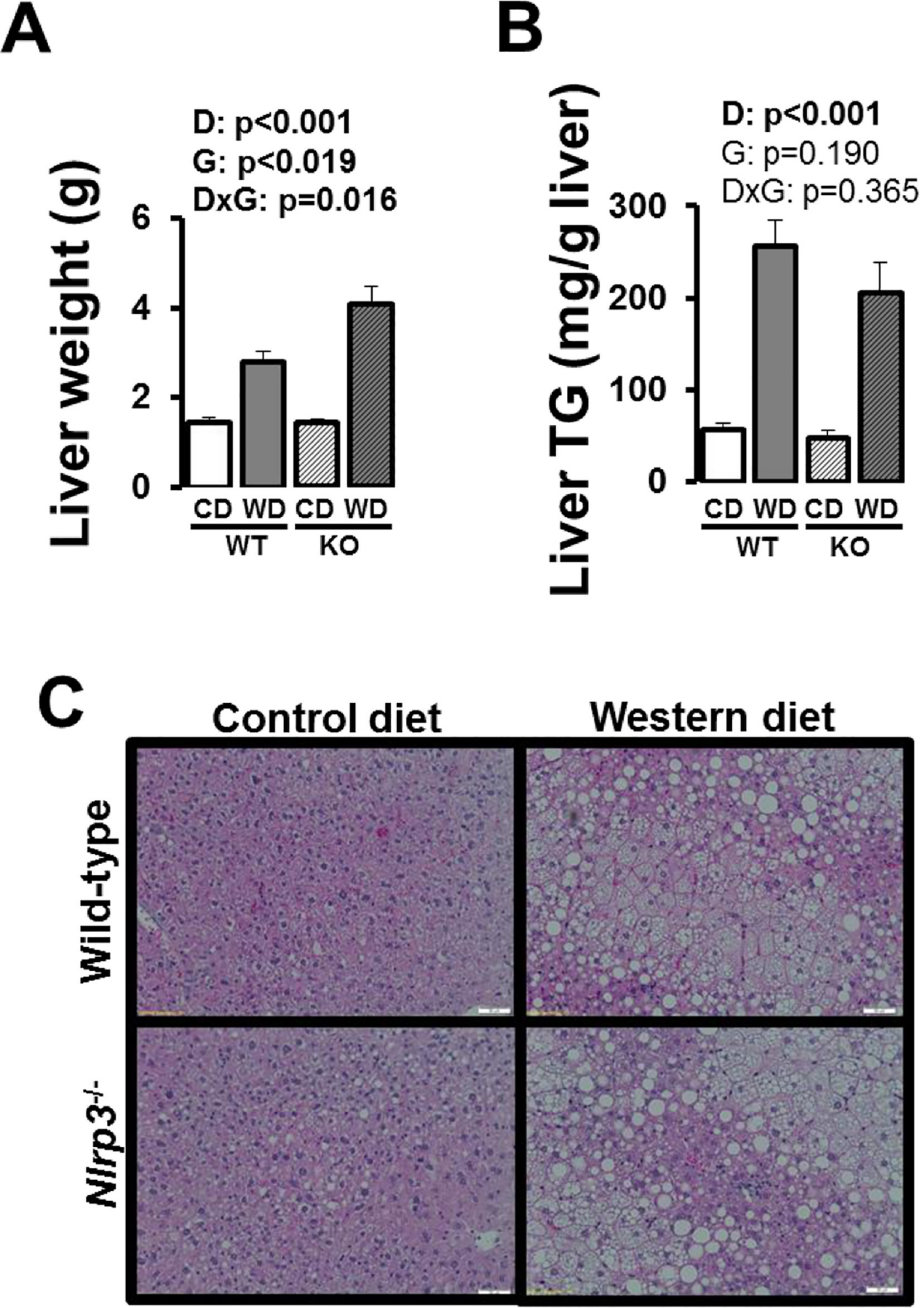
Liver characterization in WT and *Nlrp3*^-/-^ mice fed a control diet versus Western diet. (A) Liver weight; (B) liver triglycerides (TG); representative histological 20X images stained for H&E. Data are expressed as means ± SE. WT, wild-type; KO, *Nlrp3* knockout, CD, control diet; WD, Western diet; D, main effect of diet; G, main effect of genotype; DxG, diet by genotype interaction. Significant p values (<0.05) are highlighted in bold.

**Fig 5:**
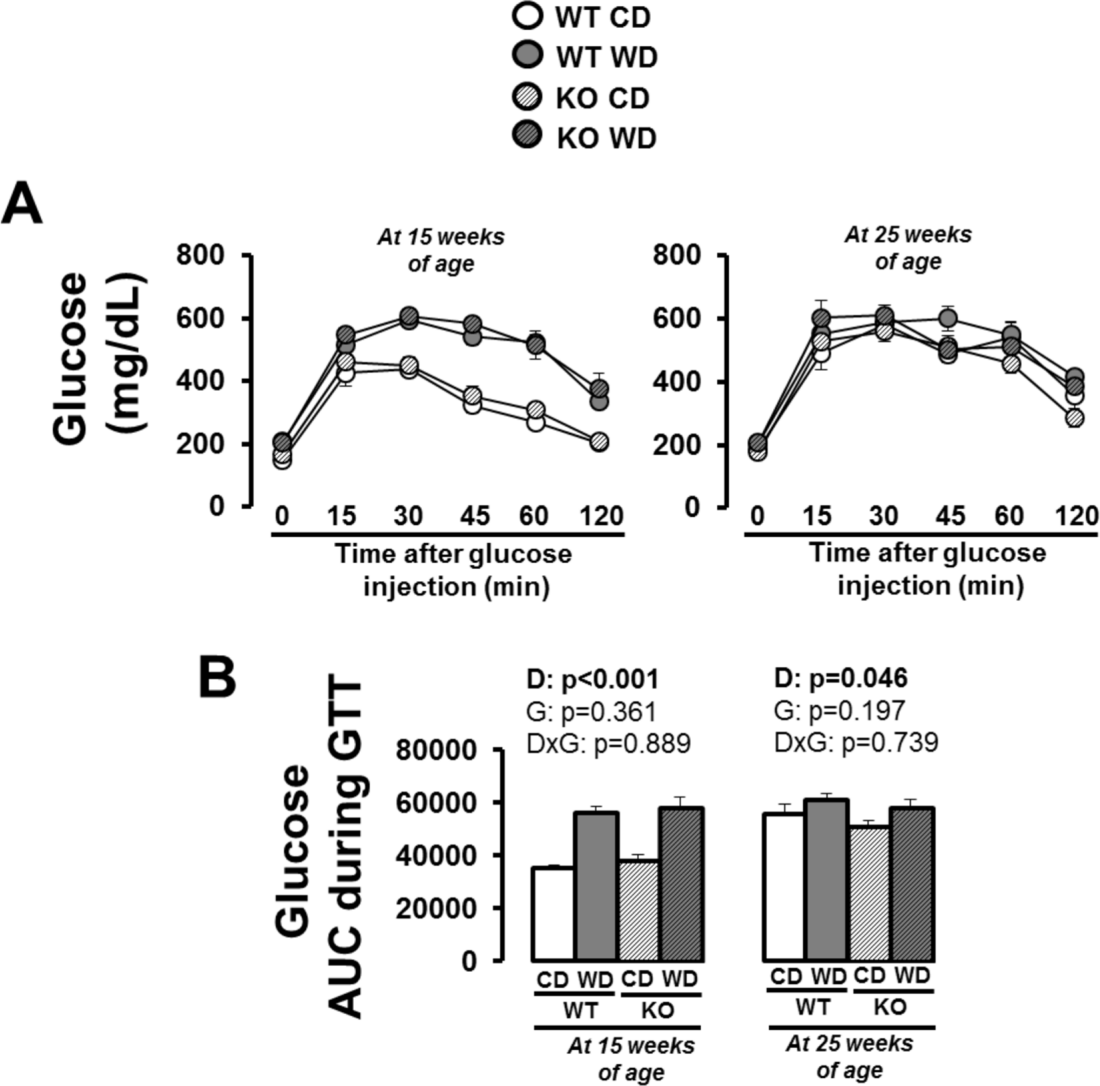
Glucose tolerance testing (GTT) in WT and *Nlrp3*^-/-^ mice fed a control diet versus Western diet at 15 and 25 weeks of age. (A) Glucose levels after glucose injection; (B) glucose area under the curve (AUC) during GTT. Data are expressed as means ± SE. WT, wild-type; KO, *Nlrp3* knockout, CD, control diet; WD, Western diet; D, main effect of diet; G, main effect of genotype; DxG, diet by genotype interaction. Significant p values (<0.05) are highlighted in bold.

Western diet increased liver weight in both WT and *Nlrp3* null mice and this increase was greater in the latter (Fig 4A; interaction p<0.05). Liver triglycerides were similarly increased in both WT and knockout mice (Fig 4B; p<0.05). Fig 5 depicts results from glucose tolerance testing. Glucose tolerance was impaired with Western diet at both time points, independent of genotype; however, the impairment was more apparent at 15 weeks of age. That is, mice fed a control diet, but not Western diet, became more glucose intolerant overtime, such that differences between control and Western diet at age 25 were less pronounced.

**Fig 6:**
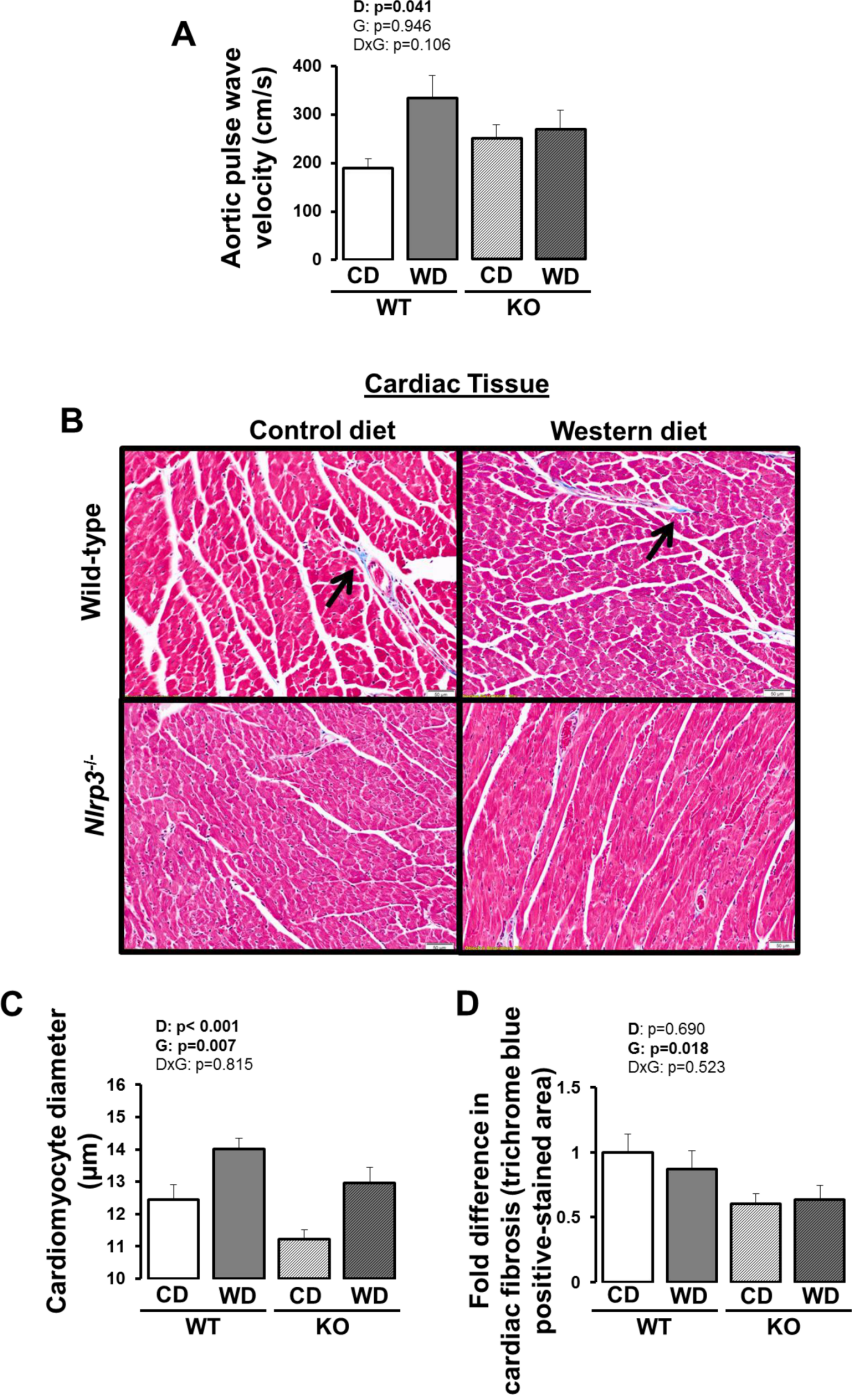
Aortic stiffness and cardiac characterization in WT and *Nlrp3*^-/-^ mice fed a control diet versus Western diet. (A) Aortic pulse wave velocity; (B) representative histological cardiac 10X images stained for trichrome blue (arrows point to trichrome blue positive-stained regions); (C) cardiomyocyte diameter; (D) cardiac fibrosis (quantification of trichrome blue positive-stained area). Data are expressed as means ± SE. WT, wild-type; KO, *Nlrp3* knockout, CD, control diet; WD, Western diet; D, main effect of diet; G, main effect of genotype; DxG, diet by genotype interaction. Significant p values (<0.05) are highlighted in bold.

Aortic PWV, a marker of arterial stiffness, was increased with Western diet in WT mice (Fig 6A; p<0.05 for pairwise comparison between control and Western diets in WT mice). In contrast, *Nlrp3*^-/-^ mice did not exhibit an increase in aortic PWV with Western diet (p>0.05), such that the diet by genotype interaction effect was trending (p = 0.106). Western diet produced an increase in cardiomyocyte size in both WT and knockout mice (Fig 6C; p<0.05). However, the cardiomyocyte size was less in *Nlrp3*^-/-^ mice compared to WT mice, independent of diet (Fig 5; p<0.05) (i.e., significant main effect of diet and genotype without diet by genotype interaction). Furthermore, cardiac fibrosis was less in *Nlrp3*^-/-^ mice compared to WT mice, independent of diet (Fig 6D; p<0.05) (i.e., significant main effect of genotype without diet by genotype interaction).

## Discussion

Counter to our hypotheses, we found that Western diet-induced white AT inflammation was not accompanied by increased caspase-1 cleavage or increased circulating levels of IL-18, both characteristic features of NLRP3 inflammasome activation. Furthermore, AT inflammation and glucose intolerance caused by Western diet were not attenuated in *Nlrp3*^-/-^ mice. However, *Nlrp3*^-/-^ mice were protected against Western diet-induced aortic stiffening, exhibited smaller cardiomyocytes, and had reduced cardiac fibrosis compared to WT mice, under both dietary conditions. Collectively, these findings suggest that presence of the *Nlrp3* gene is not required for Western diet-induced AT inflammation and glucose intolerance; yet, *Nlrp3* appears to play a role in regulating arterial stiffening, cardiac hypertrophy and fibrosis. This finding is important because it identifies a role for *Nlrp3* in the etiology of Western diet-induced cardiovascular impairments that is independent of AT or even systemic metabolic health.

### AT Inflammation Caused by Western Diet is Not Accompanied by Increased Casapse-1 Cleavage and it is Not Attenuated with Loss of Nlrp3

Chronic low-grade systemic inflammation, or metaflammation, underlies the pathogenesis of obesity-associated insulin resistance and cardiovascular disease [2–5, 22]. An important source of inflammatory cytokines is AT [2, 3]. AT expansion with caloric excess leads to infiltration of immune cells into AT, exacerbating AT inflammation and, consequently, increasing the secretion of inflammatory cytokines from AT [4, 6–8]. A better understanding of molecular mechanisms regulating AT inflammation is important as it may elucidate new therapeutic targets. Herein, we tested the hypothesis that NLRP3 inflammasome is involved in the regulation of AT inflammation. However, contrary to this notion, we found that 24 weeks of Western diet-induced expression of visceral white AT inflammatory markers was not accompanied by increased caspase-1 cleavage, a marker of NLRP3 activation. Furthermore, *Nlrp3* null mice were not protected from Western diet-induced white AT inflammation relative to WT mice. To determine if the lack of effect of *Nlrp3* deletion on AT inflammation was limited to white fat, we examined a subset of the same genes in interscapular brown AT. Similar to white AT, we found no evidence of reduced inflammation in brown AT from *Nlrp3*^-/-^ mice. Together, these findings indicate that *Nlrp3* gene is not required for Western diet-induced AT inflammation, suggesting that other mechanisms, independent of *Nlrp3*, mediate AT inflammation with obesity.

### Glucose Intolerance Caused by Western Diet is Not Attenuated with Loss of Nlrp3

AT inflammation is an important determinant of whole body insulin resistance. Accordingly, given that lack of *Nlrp3* did not abrogate Western diet-induced AT inflammation, it is not entirely surprising that glucose intolerance and increased fasting HOMA-IR, both indicators of insulin resistance, were not reduced in *Nlrp3*^-/-^ mice relative to WT mice. This finding that ablation of *Nlrp3* did not protect mice from obesity-induced insulin resistance is in contrast with other reports [23]. Vandanmagsar et al. [23] found that *Nlrp3*^-/-^ mice fed a 60% high-fat diet (HFD) exhibited lower insulin resistance, as assessed by insulin and glucose tolerance testing, than WT mice fed the same HFD. While it is difficult to reconcile this divergence in findings, it is possible this is partly attributable to the differences in diets used to promote obesity and insulin resistance. Perhaps a critical inflammatory threshold is needed for loss of *Nlrp3* to produce a phenotype in AT. In other words, the 60% HFD likely produced more inflammation and, at that critical level, the protective effect of loss of Nlrp3 could be appreciated. Alternatively, it is possible that increased whole body insulin sensitivity with elimination of *Nlrp3* only manifests when mice are kept in a specific-pathogen free barrier facility. As described [24], these facilities involve ventilated cage racks that deliver HEPA filtered air to each cage with free access to sterile water through a hydropac system. Along these lines, under conventional (i.e., non-specific pathogen free) animal housing facilities, it has been shown that inflammasome deficiency-related changes in the composition of the gut microbiota (i.e., dysbiosis) leads to exacerbated hepatic steatosis as well as multiple other aspects of metabolic syndrome such as weight gain and impaired glucose homeostasis [25]. This counterintuitive increased progression of non-alcoholic steatophepatitis reported in *Nlrp3*^-/-^ mice is even transmissible to WT mice that are co-housed with *Nlrp3*^-/-^ mice [25]. While in our study gut microbiota was not examined, it remains possible that *Nlrp3* deficiency-associated dysbiosis resulted in abnormal accumulation of bacterial products in the portal circulation, putting the liver at risk. In support of that hypothesis, we showed that plasma levels of ALT, a maker of liver damage, were 48% higher in Western diet fed *Nlrp3*^-/-^ mice compared to WT counterparts. Further, liver weights of Western diet-fed *Nlrp3*^-/-^ mice were 39% heavier than those of WT mice. Although blood and liver levels of endotoxin were not measured in this study, the increase in liver mass did not appear to be explained by increased lipid deposition as liver TGs were not different between *Nlrp3*^-/-^ and WT mice (Fig 4C). This disconnect between liver weight and lipid accumulation is intriguing and requires further investigation. However, given prior evidence that *Nlrp3*-deficient mice show accelerated clearance of triglycerides after a fat challenge [26], we speculate that the disconnect between liver weight and triglycerides may be related to an accelerated clearance of triglycerides in Nlrp3-/- mice.

As expected, *Nlrp3*^-/-^ mice demonstrated lower circulating levels of IL-18 than WT mice. The extent of reduction was comparable to that of some studies [27] but considerably less to that of others [23] that reported the blood IL-18 levels of *Nlrp3*^*-/-*^ mice to be below the detection limit of the assay. We do not have a good explanation for these discrepancies among studies; however, it is possible that differences in assay methods play a role. Regardless, it is suggestive that circulating levels of IL-18 are not exclusively regulated by *Nlrp3* gene. Given that IL-18 null mice exhibit hyperphagia, obesity and insulin resistance [28], the reduced levels of IL-18 may explain the observation that *Nlrp3*^-/-^ mice fed a Western diet appeared to be more hyperphagic and tended to be heavier than WT mice. In this regard, there is evidence that expression of leptin receptor parallels the expression of *Nlrp3* [29]. Additional studies are needed to determine if leptin signaling in the brain is reduced with loss of *Nlrp3* and whether this function of *Nlrp3* is inflammasome-independent [26, 30]. Taken together, it is possible that our finding that ablation of *Nlrp3* did not protect mice from glucose intolerance may be attributable to counteracting effects from the gut in a setting of standard co-housing facilities and/or reduced IL-18 signaling. Despite this, we did find a small, but statistically significant, reduction in levels of HbA1c in *Nlrp3*^-/-^ mice compared to WT, independent of diet. We also noted that *Nlrp3*^-/-^ mice fed a Western diet had slightly greater fasting insulin levels compared to WT mice (p = 0.19), suggesting a potential increase in β-cell function necessary for maintenance of glucose homeostasis.

### Western Diet-Induced Aortic Stiffening is Abrogated with Loss of Nlrp3

Although *Nlrp3* deficiency did not affect AT inflammation and/or glucose intolerance, we did observe that *Nlrp3*^-/-^ mice were protected from a Western diet-induced increase in aortic stiffness. This finding is relevant in light of evidence from humans indicating that aortic stiffness is an independent predictor of cardiovascular disease and all-cause mortality [31–33]. The protective effects of *Nlrp3* deficiency on the vasculature has also been suggested by others. Duewell et al. [27] found that LDL-R deficient mice whose bone marrow-derived cells lacked *Nlrp3* were resistant to the development of HFD-induced atherosclerosis. In addition, Bando et al. [34] recently showed in a cohort of 72 patients that expression of *Nlrp3* in subcutaneous white AT was an independent predictor for the severity of coronary atherosclerosis. Of interest, the authors noted that AT expression of *Nlrp3* was also associated with serum levels of uric acid [34], a likely contributor to arterial stiffness [35]. Given the growing evidence that aortic stiffness is an important determinant of cardiovascular disease and mortality [31–33], elucidating molecular mechanisms and targets for arterial de-stiffening is of paramount importance. More research is needed to examine vascular effects of *Nlrp3* signaling in the setting of obesity and metabolic disease.

### Loss of Nlrp3 Reduces Cardiac Hypertrophy and Fibrosis

Another interesting finding of the present study was the observation that *Nlrp3* null mice exhibited smaller cardiomyocytes, unlike adipocytes, and reduced cardiac fibrosis in control and Western diets. This finding is relevant in that cardiac hypertrophy and fibrosis contribute to the progression of heart failure. In this regard, Bracey et al. [36] recently showed in mice that cardiac-specific overexpression of the calcineurin transgene (CNTg), a model of heart failure, resulted in increased cardiac expression of *Nlrp3*, hypertrophy and reduced fractional shortening, indicative of cardiac dysfunction. Importantly, CNTg mice bred into the *Nlrp3*^-/-^ background exhibited restored cardiac function [36]. There is also evidence in a rat model of type 2 diabetes that *Nlrp3* gene silencing ameliorates cardiac inflammation, fibrosis, and function and that NLRP3 inflammasome activation is mediated by increased oxidative stress-induced NF-kB activation [37]. Also, it has been reported that *Nlrp3*-deficient mice are protected against angiotensin II-induced cardiac fibrosis [38] and that pharmacological inhibition of NLRP3 markedly reduces cardiac fibrosis and preserves systolic function after ischemic and nonischemic injuries in mice [39]. The role of *Nlrp3* in regulating fibrosis has also been reported in non-cardiac tissues including the kidney. For example, it has been shown that deletion of *Nlrp3* protects renal fibrosis via inhibition of mitochondrial dysfunction [40]. These findings, combined with our data, support the role of *Nlrp3* in modulating cardiac structure and function and highlight the importance of *Nlrp3* as a potential therapeutic target. Although more studies are necessary to elucidate the underlying mechanisms by which *Nlrp3* regulates collagen synthesis, fibrosis, hypertrophy, and cardiac function, we speculate that mitochondrial dysfunction and oxidative stress may be implicated in these processes. Specifically, it is possible that loss of *Nlrp3* mitigates mitochondrial-derived production of reactive oxygen species. Because reactive oxygen species are a signal for collagen synthesis, fibrosis, and cardiovascular stiffness [41–43], the cardiovascular protection in *Nlrp3* null mice may be in part attributed to reduced oxidative stress.

### Considerations

Several considerations for the overall interpretation of the current findings are warranted. First, in our study, we found that 24 weeks of Western diet feeding did not significantly increase caspase-1 cleavage in AT or circulating levels of IL-18, two markers of NLRP3 inflammasome activation. Given that activated caspase-1 is secreted from cells as a function of time, it is possible that we missed the window of time during which p10 casapse-1 was increased in AT. That is, at this time, we cannot rule out the possibility that after 24 weeks of Western diet feeding caspase-1 processing and secretion may be increased. Therefore, the need for additional time-course studies evaluating the kinetics of caspase-1 cleavage and secretion from AT during Western diet would be of interest. Second, AT inflammation was assessed via RT-PCR (in whole tissue and stromal vascular cells), immunohistochemically with Mac-2 staining, and by Western blotting (Mac-2 protein content) in whole tissue. Future research using FACS analysis is needed to examine the differential inflammatory effects of obesity and *Nlrp3* ablation in AT immune cells (i.e., macrophages, T-cells) vs. adipocytes. Third, while it has been described that *Nlrp3* deficiency-associated dysbiosis leads to liver complications and impaired glucose homeostasis [25], our study did not characterize the microbiota or endotoxin levels, which can be considered a limitation. The mechanisms by which *Nlrp3* appear to play a role in cardiovascular tissue, and its implications, remain largely elusive. Accordingly, additional studies should further characterize the cardiovascular phenotype of *Nlrp3* null mice at the molecular and functional levels including measures of blood pressure, endothelial function, and cardiac function. It should also be noted that this study was conducted in male mice undergoing a 24-week control vs. Western diet intervention, kept in a non-specific pathogen free facility, and fasted for 5 hours prior to tissue harvest. In short, future studies need to establish the extent to which differences in sex, duration and composition of diets, facility conditions, and hours of fasting have an impact on these results.

### Conclusion

In conclusion, findings from the present study indicate that Western diet-induced glucose intolerance and AT inflammation is *Nlrp3*-independent. Nonetheless, we showed that *Nlrp3* is involved in the regulation of arterial stiffening and cardiac remodeling, suggesting that *Nlrp3* may be a therapeutic target for the prevention and treatment of cardiovascular disease. More research is needed to understand the physiological role of *Nlrp3* across organs, as very recently put forth in a review [44].

## References

[1] Farb MG, Gokce N. Visceral adiposopathy: a vascular perspective. Hormone molecular biology and clinical investigation. 2015;21(2):125–36. Epub 2015/03/18. 10.1515/hmbci-2014-0047 ; PubMed Central PMCID: PMCPmc4442778. 25781557 PMC4442778

[2] Reaven GM. Insulin resistance: the link between obesity and cardiovascular disease. The Medical clinics of North America. 2011;95(5):875–92. Epub 2011/08/23. 10.1016/j.mcna.2011.06.002 . 21855697

[3] Ginsberg HN, MacCallum PR. The obesity, metabolic syndrome, and type 2 diabetes mellitus pandemic: Part I. Increased cardiovascular disease risk and the importance of atherogenic dyslipidemia in persons with the metabolic syndrome and type 2 diabetes mellitus. Journal of the cardiometabolic syndrome. 2009;4(2):113–9. Epub 2009/07/21. 10.1111/j.1559-4572.2008.00044.x ; PubMed Central PMCID: PMCPmc2901596. 19614799 PMC2901596

[4] Vieira-Potter VJ. Inflammation and macrophage modulation in adipose tissues. Cellular microbiology. 2014;16(10):1484–92. Epub 2014/07/31. 10.1111/cmi.12336 . 25073615

[5] Kanda H, Tateya S, Tamori Y, Kotani K, Hiasa K, Kitazawa R, et al MCP-1 contributes to macrophage infiltration into adipose tissue, insulin resistance, and hepatic steatosis in obesity. J Clin Invest. 2006;116(6):1494–505. Epub 2006/05/13. 10.1172/jci26498 ; PubMed Central PMCID: PMCPmc1459069. 16691291 PMC1459069

[6] Grant RW, Stephens JM. Fat in flames: influence of cytokines and pattern recognition receptors on adipocyte lipolysis. Journal Article. 2015;309(3):E205–13. Epub 2015/06/11. 10.1152/ajpendo.00053.2015 . 26058863

[7] Jacobi D, Stanya KJ, Lee CH. Adipose tissue signaling by nuclear receptors in metabolic complications of obesity. Adipocyte. 2012;1(1):4–12. Epub 2012/08/24. 10.4161/adip.19036 ; PubMed Central PMCID: PMCPmc3423221. 22916336 PMC3423221

[8] Vieira-Potter VJ, Zidon TM, Padilla J. Exercise and Estrogen Make Fat Cells "Fit". Exercise and sport sciences reviews. 2015;43(3):172–8. Epub 2015/04/24. 10.1249/jes.0000000000000046 . 25906425 PMC4950919

[9] Grant RW, Dixit VD. Mechanisms of disease: inflammasome activation and the development of type 2 diabetes. Frontiers in immunology. 2013;4:50 Epub 2013/03/14. 10.3389/fimmu.2013.00050 ; PubMed Central PMCID: PMCPmc3592198. 23483669 PMC3592198

[10] Grant RW, Dixit VD. Adipose tissue as an immunological organ. Obesity (Silver Spring). 2015;23(3):512–8. Epub 2015/01/23. 10.1002/oby.21003 ; PubMed Central PMCID: PMCPmc4340740. 25612251 PMC4340740

[11] Dixit VD. Nlrp3 inflammasome activation in type 2 diabetes: is it clinically relevant? Diabetes. 2013;62(1):22–4. Epub 2012/12/22. 10.2337/db12-1115 ; PubMed Central PMCID: PMCPmc3526045. 23258906 PMC3526045

[12] Mori MA, Bezy O, Kahn CR. Metabolic syndrome: is Nlrp3 inflammasome a trigger or a target of insulin resistance? Circulation research. 2011;108(10):1160–2. Epub 2011/05/14. 10.1161/RES.0b013e318220b57b ; PubMed Central PMCID: PMCPmc3660550. 21566220 PMC3660550

[13] De Nardo D, Latz E. NLRP3 inflammasomes link inflammation and metabolic disease. Trends in immunology. 2011;32(8):373–9. Epub 2011/07/08. 10.1016/j.it.2011.05.004 ; PubMed Central PMCID: PMCPmc3151541. 21733753 PMC3151541

[14] Pettersson US, Walden TB, Carlsson PO, Jansson L, Phillipson M. Female mice are protected against high-fat diet induced metabolic syndrome and increase the regulatory T cell population in adipose tissue. PLoS One. 2012;7(9):e46057 Epub 2012/10/11. 10.1371/journal.pone.0046057 ; PubMed Central PMCID: PMCPmc3458106. 23049932 PMC3458106

[15] Vieira-Potter VJ, Padilla J, Park YM, Welly RJ, Scroggins RJ, Britton SL, et al Female rats selectively bred for high intrinsic aerobic fitness are protected from ovariectomy-associated metabolic dysfunction. American journal of physiology Regulatory, integrative and comparative physiology. 2015;308(6):R530–42. Epub 2015/01/23. 10.1152/ajpregu.00401.2014 ; PubMed Central PMCID: PMCPmc4360065. 25608751 PMC4360065

[16] Kaiyala KJ, Morton GJ, Leroux BG, Ogimoto K, Wisse B, Schwartz MW. Identification of body fat mass as a major determinant of metabolic rate in mice. Diabetes. 2010;59(7):1657–66. Epub 2010/04/24. 10.2337/db09-1582 ; PubMed Central PMCID: PMCPmc2889765. 20413511 PMC2889765

[17] DeMarco VG, Habibi J, Jia G, Aroor AR, Ramirez-Perez FI, Martinez-Lemus LA, et al Low-Dose Mineralocorticoid Receptor Blockade Prevents Western Diet-Induced Arterial Stiffening in Female Mice. Hypertension. 2015;66(1):99–107. Epub 2015/05/28. 10.1161/hypertensionaha.115.05674 ; PubMed Central PMCID: PMCPmc4465849. 26015449 PMC4465849

[18] Wainright KS, Fleming NJ, Rowles JL, Welly RJ, Zidon TM, Park YM, et al Retention of sedentary obese visceral white adipose tissue phenotype with intermittent physical activity despite reduced adiposity. American journal of physiology Regulatory, integrative and comparative physiology. 2015;309(5):R594–602. Epub 2015/07/17. 10.1152/ajpregu.00042.2015 ; PubMed Central PMCID: PMCPmc4591377. 26180183 PMC4591377

[19] Coelho-Filho OR, Shah RV, Mitchell R, Neilan TG, Moreno H Jr., Simonson B, et al Quantification of cardiomyocyte hypertrophy by cardiac magnetic resonance: implications for early cardiac remodeling. Circulation. 2013;128(11):1225–33. Epub 2013/08/06. 10.1161/circulationaha.112.000438 . 23912910 PMC5308548

[20] Matyas C, Nemeth BT, Olah A, Hidi L, Birtalan E, Kellermayer D, et al The soluble guanylate cyclase activator cinaciguat prevents cardiac dysfunction in a rat model of type-1 diabetes mellitus. Cardiovasc Diabetol. 2015;14(1):145 Epub 2015/11/02. 10.1186/s12933-015-0309-x ; PubMed Central PMCID: PMCPmc4628236. 26520063 PMC4628236

[21] Vieira Potter V, Strissel KJ, Xie C, Chang E, Bennett G, Defuria J, et al Adipose tissue inflammation and reduced insulin sensitivity in ovariectomized mice occurs in the absence of increased adiposity. Endocrinology. 2012;153(9):4266–77. 10.1210/en.2011-2006 22778213 PMC3423617

[22] Patel MN, Bernard WG, Milev NB, Cawthorn WP, Figg N, Hart D, et al Hematopoietic IKBKE limits the chronicity of inflammasome priming and metaflammation. 2015;112(2):506–11. 10.1073/pnas.1414536112 . 25540417 PMC4299251

[23] Vandanmagsar B, Youm YH, Ravussin A, Galgani JE, Stadler K, Mynatt RL, et al The NLRP3 inflammasome instigates obesity-induced inflammation and insulin resistance. Nature medicine. 2011;17(2):179–88. Epub 2011/01/11. 10.1038/nm.2279 ; PubMed Central PMCID: PMCPmc3076025. 21217695 PMC3076025

[24] Youm YH, Grant RW, McCabe LR, Albarado DC, Nguyen KY, Ravussin A, et al Canonical Nlrp3 inflammasome links systemic low-grade inflammation to functional decline in aging. Cell metabolism. 2013;18(4):519–32. Epub 2013/10/08. 10.1016/j.cmet.2013.09.010 ; PubMed Central PMCID: PMCPmc4017327. 24093676 PMC4017327

[25] Henao-Mejia J, Elinav E, Jin C, Hao L, Mehal WZ, Strowig T, et al Inflammasome-mediated dysbiosis regulates progression of NAFLD and obesity. Nature. 2012;482(7384):179–85. Epub 2012/02/03. 10.1038/nature10809 ; PubMed Central PMCID: PMCPmc3276682. 22297845 PMC3276682

[26] Kotas ME, Jurczak MJ, Annicelli C, Gillum MP, Cline GW, Shulman GI, et al Role of caspase-1 in regulation of triglyceride metabolism. Proceedings of the National Academy of Sciences of the United States of America. 2013;110(12):4810–5. Epub 2013/03/15. 10.1073/pnas.1301996110 ; PubMed Central PMCID: PMCPmc3607017. 23487794 PMC3607017

[27] Duewell P, Kono H, Rayner KJ, Sirois CM, Vladimer G, Bauernfeind FG, et al NLRP3 inflammasomes are required for atherogenesis and activated by cholesterol crystals. Nature. 2010;464(7293):1357–61. Epub 2010/04/30. 10.1038/nature08938 ; PubMed Central PMCID: PMCPmc2946640. 20428172 PMC2946640

[28] Netea MG, Joosten LA, Lewis E, Jensen DR, Voshol PJ, Kullberg BJ, et al Deficiency of interleukin-18 in mice leads to hyperphagia, obesity and insulin resistance. Nature medicine. 2006;12(6):650–6. Epub 2006/05/30. 10.1038/nm1415 . 16732281

[29] Bigford GE, Bracchi-Ricard VC, Keane RW, Nash MS, Bethea JR. Neuroendocrine and cardiac metabolic dysfunction and NLRP3 inflammasome activation in adipose tissue and pancreas following chronic spinal cord injury in the mouse. ASN neuro. 2013;5(4):243–55. Epub 2013/08/09. 10.1042/an20130021 ; PubMed Central PMCID: PMCPmc3789215. 23924318 PMC3789215

[30] Inoue Y, Shirasuna K, Kimura H, Usui F, Kawashima A, Karasawa T, et al NLRP3 regulates neutrophil functions and contributes to hepatic ischemia-reperfusion injury independently of inflammasomes. Journal of immunology (Baltimore, Md: 1950). 2014;192(9):4342–51. Epub 2014/04/04. 10.4049/jimmunol.1302039 . 24696236

[31] Vlachopoulos C, Manesis E, Baou K, Papatheodoridis G, Koskinas J, Tiniakos D, et al Increased arterial stiffness and impaired endothelial function in nonalcoholic Fatty liver disease: a pilot study. Am J Hypertens. 2010;23(11):1183–9. 10.1038/ajh.2010.144 20634799

[32] Pannier B, Guerin AP, Marchais SJ, Safar ME, London GM. Stiffness of capacitive and conduit arteries: prognostic significance for end-stage renal disease patients. Hypertension. 2005;45(4):592–6. Epub 2005/03/09. 10.1161/01.HYP.0000159190.71253.c3 . 15753232

[33] Mitchell GF, Hwang SJ, Vasan RS, Larson MG, Pencina MJ, Hamburg NM, et al Arterial stiffness and cardiovascular events: the Framingham Heart Study. Circulation. 2010;121(4):505–11. Epub 2010/01/20. 10.1161/circulationaha.109.886655 ; PubMed Central PMCID: PMCPmc2836717. 20083680 PMC2836717

[34] Bando S, Fukuda D, Soeki T, Nishimoto S, Uematsu E, Matsuura T, et al Expression of NLRP3 in subcutaneous adipose tissue is associated with coronary atherosclerosis. Atherosclerosis. 2015;242(2):407–14. Epub 2015/08/19. 10.1016/j.atherosclerosis.2015.07.043 . 26282945

[35] Mehta T, Nuccio E, McFann K, Madero M, Sarnak MJ, Jalal D. Association of Uric Acid With Vascular Stiffness in the Framingham Heart Study. Am J Hypertens. 2015;28(7):877–83. Epub 2015/01/02. 10.1093/ajh/hpu253 ; PubMed Central PMCID: PMCPmc4542908. 25552515 PMC4542908

[36] Bracey NA, Beck PL, Muruve DA, Hirota SA, Guo J, Jabagi H, et al The Nlrp3 inflammasome promotes myocardial dysfunction in structural cardiomyopathy through interleukin-1beta. Experimental physiology. 2013;98(2):462–72. Epub 2012/08/01. 10.1113/expphysiol.2012.068338 . 22848083

[37] Luo B, Li B, Wang W, Liu X, Xia Y, Zhang C, et al NLRP3 gene silencing ameliorates diabetic cardiomyopathy in a type 2 diabetes rat model. PLoS One. 2014;9(8):e104771 Epub 2014/08/20. 10.1371/journal.pone.0104771 ; PubMed Central PMCID: PMCPmc4138036. 25136835 PMC4138036

[38] Bracey NA, Gershkovich B, Chun J, Vilaysane A, Meijndert HC, Wright JR Jr., et al Mitochondrial NLRP3 protein induces reactive oxygen species to promote Smad protein signaling and fibrosis independent from the inflammasome. The Journal of biological chemistry. 2014;289(28):19571–84. Epub 2014/05/21. 10.1074/jbc.M114.550624 ; PubMed Central PMCID: PMCPmc4094069. 24841199 PMC4094069

[39] Marchetti C, Toldo S, Chojnacki J, Mezzaroma E, Liu K, Salloum FN, et al Pharmacologic Inhibition of the NLRP3 Inflammasome Preserves Cardiac Function After Ischemic and Nonischemic Injury in the Mouse. J Cardiovasc Pharmacol. 2015;66(1):1–8. Epub 2015/04/29. 10.1097/fjc.0000000000000247 ; PubMed Central PMCID: PMCPmc4500673. 25915511 PMC4500673

[40] Gong W, Mao S, Yu J, Song J, Jia Z, Huang S, et al NLRP3 Deletion Protects against Renal Fibrosis and Attenuates Mitochondrial Abnormality in Mouse with 5/6 Nephrectomy. American journal of physiology Renal physiology. 2016:ajprenal.00534.2015. Epub 2016/02/19. 10.1152/ajprenal.00534.2015 . 26887832

[41] Kayama Y, Raaz U, Jagger A, Adam M, Schellinger IN, Sakamoto M, et al Diabetic Cardiovascular Disease Induced by Oxidative Stress. International journal of molecular sciences. 2015;16(10):25234–63. Epub 2015/10/30. 10.3390/ijms161025234 ; PubMed Central PMCID: PMCPmc4632800. 26512646 PMC4632800

[42] Jia G, Habibi J, Bostick BP, Ma L, DeMarco VG, Aroor AR, et al Uric acid promotes left ventricular diastolic dysfunction in mice fed a Western diet. Hypertension. 2015;65(3):531–9. Epub 2014/12/10. 10.1161/hypertensionaha.114.04737 ; PubMed Central PMCID: PMCPmc4370431. 25489061 PMC4370431

[43] Jia G, DeMarco VG, Sowers JR. Insulin resistance and hyperinsulinaemia in diabetic cardiomyopathy. Nature reviews Endocrinology. 2016;12(3):144–53. Epub 2015/12/19. 10.1038/nrendo.2015.216 ; PubMed Central PMCID: PMCPmc4753054. 26678809 PMC4753054

[44] Coll R, O'Neill L, Schroder K. Questions and controversies in innate immune system reserach: what is the physiological role of NLRP3? Cell Death Discovery. 2016;2(16019). 10.1038/cddiscovery.2016.19 PMC4979470 27551512

